# Satisfaction with a digitally-enabled telephone health coaching intervention for people with non-diabetic hyperglycaemia

**DOI:** 10.1038/s41746-019-0080-6

**Published:** 2019-02-04

**Authors:** Peter Coventry, Peter Bower, Amy Blakemore, Elizabeth Baker, Mark Hann, Jinshuo Li, Angela Paisley, Martin Gibson

**Affiliations:** 1 Department of Health Sciences and Centre for Reviews and Dissemination, University of York, York, UK; 2 NIHR School for Primary Care Research, Centre for Primary Care, Manchester Academic Health Science Centre, University of Manchester, Manchester, UK; 3 Division of Nursing, Midwifery and Social Work, School of Health Sciences, University of Manchester, Manchester, UK; 4 Department of Health Sciences, University of York, York, UK; 5 Salford Royal NHS Foundation Trust, Salford, UK

**Keywords:** Health care, Risk factors

## Abstract

International evidence shows that lifestyle interventions can effectively reduce the risk of developing diabetes in people with non-diabetic hyperglycaemia (NDH). A candidate intervention that has potential to be rolled out at population level is health coaching. Digital interventions offer the means to potentially enhance user satisfaction with health coaching and improve efficiencies. We used a randomised controlled trial to test whether a digitally-enabled health coaching intervention that included an online dashboard and telephone health coaching improved user satisfaction and cost-efficiencies compared with a telephone only health coaching intervention. The primary outcome was satisfaction measured by Client Satisfaction Questionnaire (CSQ-8). 103 participants with NDH were allocated to the telephone coaching only intervention and 106 participants with NDH were allocated to the digital and telephone coaching intervention. In an intention-to-treat analysis satisfaction was higher in participants allocated to the digital and telephone coaching intervention than those allocated to the telephone only intervention, but the difference was not significant. There were no significant differences between the groups on secondary outcomes (HbA1c, BMI, activation, depression, self-management, health status). From a service commissioning perspective the mean incremental cost of the digitally-enabled intervention was £236 ($332; €270). Call times, including administration, were longer for participants allocated to the digitally-enabled intervention. The results show that user satisfaction with digitally-enabled intervention is broadly equivalent with that of telephone delivered interventions in the context of routinely delivered diabetes prevention programmes. There is scope for future work that assesses how economies of scale can be achieved at larger user bases.

## Introduction

Diabetes is a major global public health threat affecting nearly half a billion adults.^1^ Over 90% of cases are type 2 diabetes which develops in the presence of genetic, environmental and behavioural factors; the majority of cases are attributable to excess body weight and sedentary lifestyles.^2^ Unmanaged, type 2 diabetes is associated with serious microvascular and macrovascular complications that can lead to significant disability and premature mortality.^3^ The cost implications to health systems is considerable: the total cost of diabetes care in the UK National Health Service (NHS) was £23.7 billion ($33.8 bn, €27.4 bn) in 2010–11, with a projected annual cost of £39.8 bn ($56.8 bn, €46 bn) by 2035–36.^4^

The onset of these complications can occur during a latent or pre-diabetic phase characterised by impaired glucose metabolism with fasting plasma glucose levels below internationally agreed thresholds for diabetes. Non-diabetic hyperglycaemia (NDH) is a high-risk category of developing diabetes and is a term used to describe pre-diabetic conditions associated with decreased ability of the body to regulate glucose effectively, such as impaired glucose regulation (IGR) and impaired fasting glucose and/or impaired glucose tolerance.^5^

There is unequivocal evidence that lifestyle interventions can reduce the risk of developing diabetes.^6^ When targeted at people with NDH, lifestyle interventions that promote physical activity, diet modification and weight loss can reduce the risk of diabetes by 58%.^7,8^ However, while the use of lifestyle interventions to prevent diabetes in people with NDH are recommended in national guidelines, they are usually resource intensive to deliver and are unlikely to be cost-effective and implementable at scale across routine settings.^9^

Identifying cost-effective lifestyle interventions that can be deployed in routine settings to prevent diabetes in high risk populations such as those with NDH is therefore a health policy priority.^10^ Health coaching that includes modelling behaviour and goal setting, has emerged as a promising candidate platform that can support the delivery of effective lifestyle interventions for people with long term conditions,^11^ including diabetes^12^ and possibly people with NDH.^13^ With the flexibility of being accessible by telephone, health coaching can potentially reach a significant proportion of the target population.

It is possible that reach and efficiency of telephone health coaching could be enhanced further by digitally-enabled components. The last decade has seen increased use and availability of second generation web-based interventions that include program and interactive content, multimedia materials and guidance and feedback.^14^ Web-based interventions have been especially used to promote behaviour change and manage mental health problems.^15,16^ And there is good evidence that technology assisted primary care interventions that combine either the internet, personal computer, and/or a mobile device are effective in supporting people to lose weight.^17^ Fully automated interventions that support web-based and mobile facilitated goal setting and personalised behaviour change have also proven effective in reducing cardiovascular risk among overweight and obese adults and in improving glycaemic control and reducing other risk factors for diabetes, but gains are short-lived.^18,19^

While there is an emerging consensus about best practice in developing and evaluating effective web-based interventions, there is less emphasis on understanding how end-user satisfaction can inform successful implementation in routine settings.^20^ In the context of web-based interventions user satisfaction and experience ratings are important metrics that go beyond developer assessments of design and build quality and potentially provide critical feedback about usability and acceptability relevant to healthcare providers.^21^ Satisfaction is increasingly used as an outcome of interest in evaluations of web-based health interventions and there is growing recognition that satisfaction can capture user perception of their experience of web-based interventions.^22,23^

In the context of the CATFISH trial a telephone-only health coaching intervention for people with NDH was established as routine care and the aim of the trial was therefore to compare two active treatments to determine if a digitally-enabled health coaching intervention improved user experience and led to higher efficiency. Specifically the CATFISH trial aims to assess whether a digitally-enabled telephone coaching intervention (IGR3) is more acceptable than an existing telephone-only coaching intervention (IGR2) for people with NDH. Secondary objectives of the trial are to (1) determine whether the delivery of the IGR3 intervention is more efficient than the existing commissioned IGR2 intervention; (2) to explore the cost-effectiveness of IGR3 in comparison with IGR2; (3) to qualitatively explore and compare user and provider experience of IGR3 and IGR2 interventions; and (4) to explore the impact, if any, of IGR3 compared with IGR2 on clinical outcomes relevant to diabetes prevention in people with NDH

## Results

### Participant recruitment and retention

**Fig. 1:**
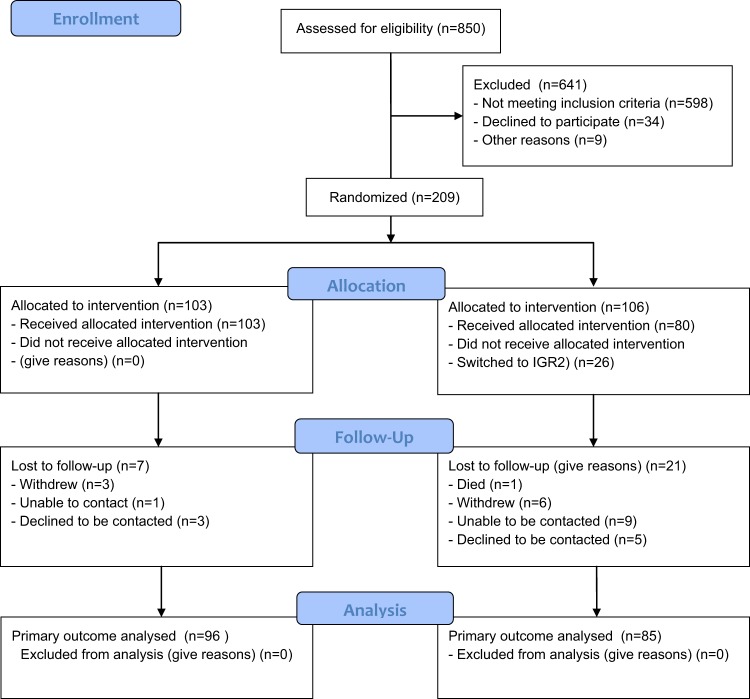
CONSORT flow diagram

Between July 2015 and May 2016 there were 853 referrals from general practice in Salford to Care Call. Of these 253 (29.7%) verbally consented to be contacted by the research team at the University of Manchester about participating in the CATFISH trial. Of those that consented to be contacted 210 (83%) agreed to take part in the CATFISH trial and were assessed for eligibility and invited to a baseline assessment following receipt of their written informed consent. The first participant was recruited on 30th June 2015 and the last participant was recruited on 25th May 2016. One participant was withdrawn from the trial before allocation because they were found to have type 2 diabetes. 209 participants were randomised, with 103 allocated to IGR2 and 106 allocated to IGR3. 87% of participants returned a follow-up questionnaire for the primary outcome. The flow of participants is shown in Fig. 1.

### Baseline characteristics of participants

Participants had a mean age of 58.3 (SD 11.4) years and 44% were female. The majority (94%) were from white ethnic backgrounds and just under half (47.5%) were either in full or part-time paid employment. Participants had a mean HbA1c of 44.4 (SD 1.6) mmol/mol, which is equivalent to 6.2% HbA1c. All participants had a BMI in the obese range (>30 Kg/m^2^) at baseline, with a mean BMI of 33.9 (SD 7.3). Over a quarter (28.2%) of participants met the threshold on MHI-5 for probable depression at baseline. Participants in the two groups were similar in all respects, except that a higher proportion of females were allocated to IGR2 compared with IGR3 (48.5 vs. 39.6%). Table 1 shows the baseline characteristics of the participants by treatment allocation.

**Table 1:** Baseline characteristics of participants

	IGR2 (*n* = 103)	IGR3 (*n* = 106)
Mean age	59.0 (11.3)	57.6 (11.6)
Age in categories:		
25–44 years	9 (8.7%)	11 (10.3%)
45–64 years	59 (57.3%)	64 (60.4%)
65–84 years	35 (34.0%)	31 (29.2%)
Sex (female)	50 (48.5%)	42 (39.6%)
Ethnicity		
White (British/Irish/Other)	97 (94.2%)	99 (93.4%)
Non-White	6 (5.8%)	7 (6.6%)
Economic status		
Working full-time	34 (33.3%)	41 (39.4%)
Working part-time	13 (12.7%)	11 (10.6%)
Unemployed	7 (6.9%)	3 (2.9%)
Permanently sick	8 (7.8%)	8 (7.7%)
Retired	34 (33.3%)	32 (30.8%)
Other (F/T Education/Looking after Home/Something Else)	6 (5.9%)	9 (8.6%
Missing data	1	2
Mean BMI	33.4 (6.7)	34.4 (8.0)
Mean HbA1c (mmol/mol)	44.7 (1.6)	44.2 (1.6)
Mean patient activation (PAM-13)	62.2 (14.3)	60.8 (13.1)
Mean general health VAS	69.3 (19.8)	66.1 (19.9)
Mean MHI score	71.9 (20.8)	70.3 (20.2)
Probable depression diagnosis		
Depression	26 (25.2%)	32 (30.2%)
No depression	77 (74.8%)	74 (69.8%)

*Note*: Data are means (SD) or numbers (%)

*BMI* body mass index, *EQ-5DL* EuroQol-5DL, *HbA1c* Glycated haemoglobin, *MHI* Mental health inventory, *mmol* millimoles per litre, *mol* mole per litre, *PAM* patient activation measure, *VAS* visual analogue scale

### Intervention uptake and adherence

Seven participants (one allocated to IGR2; six allocated to IGR3) withdrew before they received an action planning call. A further 10 participants (three allocated to IGR2; seven allocated to IGR3) did not receive any follow-up calls after the initial action planning call. Twenty-six participants allocated to IGR3 switched to IGR2 during the course of their exposure to the intervention. Ninety-nine participants in IGR2 and 93 participants in IGR3 had at least one follow-up call and the majority of these (73% in IGR2; 64% in IGR3) had more than five attempted follow-up calls. The mean total health advisor contact time per participant was 142.6 (SD 50.7) minutes for IGR2 and 132.2 (SD 66.5) minutes for IGR3.

### Outcomes

We collected primary outcome data for 181 (87%) participants. Missing outcome data were therefore imputed for 28 participants. At follow-up, the mean score on the CSQ-8 in IGR2 was 27.1 (SD 5.2), and in IGR3 was 28.4 (SD 4.5). Controlling for relevant covariates, the total score for the CSQ-8 was 1.32 points higher (95% confidence interval −0.13 to 2.77; *p* = 0.074) in participants allocated to IGR3 compared with those allocated to IGR2. This equates to a small effect size (standardised mean difference 0.29, 95% C.I. −0.01 to 0.58). There is, therefore, weak evidence in favour of IGR3, but not at the 5% level of significance. Both the per-protocol (CSQ-8 difference = 1.33; 95% C.I. −0.22 to 2.88; *p* = 0.091) and complete case (CSQ-8 difference = 1.39; 95% C.I. −0.03 to 2.88; *p* = 0.062) analyses for the primary outcome returned very similar results to the intention to treat analyses: differences in total satisfaction scores between IGR2 and IGR3 were not significant at the 5% level.

Secondary outcomes were only analysed and presented for complete cases (Table 2). While secondary outcomes broadly favoured IGR3 the differences did not reach significance. Only the between group difference in BMI was borderline significant (*p* = 0.05).

**Table 2:** Complete case analyses of secondary outcomes at 9-months follow-up

	IGR2		IGR3			
Outcome	No of participants	Mean (SD)	No of participants	Mean (SD)	Adjusted difference in means (95% CI)^a^	*P* value
HbA1c (mmol/mol)	77	43.4 (4.2)	70	42.9 (3.2)	−0.37 (−1.48 to 0.67)	0.50
BMI (kg/m^2^)	89	33.8 (6.7)	84	32.4 (7.3)	−0.52 (−1.05 to −0.00)	0.05
EQ−5D−5L	53	0.72 (0.3)	58	0.73 (0.2)	0.02 (−0.00 to 0.03)	0.07
General Health VAS (0–100)	96	71.5 (19.4)	84	73.3 (22.1)	2.86 (−2.5 to 7.9)	0.27
MHI (0–100)	94	73.0 (19.7)	85	72.7 (19.9)	0.07 (−4.0 to 4.0)	0.97
PAM score (0–100)	96	64.6 (15.3)	85	65.2 (13.8)	0.78 (−2.9 to 4.4)	0.67
SDSCA						
Days ate ≥5 portions of Fruit and vegetables	95	4.54 (1.7)	85	4.33 (1.9)		
Days ate high fat foods	96	4.35 (1.8)	85	4.26 (2.1)		
Days participated in ≥30 mins physical activity	102	4.1 (2.2)	85	4.6 (2.1)		
Days participate in specific exercise session	96	1.2 (1.7)	85	1.2 (1.8)		
Days drank alcohol	96	5.4 (2.1)	85	5.6 (1.9)		
Cigarettes per day	18	13.6 (7.6)	17	13.0 (7.8)		

*BMI*body mass index, *EQ-5DL*EuroQol-5DL, *HbA1c*glycated haemoglobin, *MHI*mental health inventory, *mmol*millimoles per litre, *mol*mole per litre, *PAM*patient activation measure, *SDSCA*summary of diabetes self-care activities, *VAS*visual analogue scale

^a^Adjusted differences are those reported by the regression models - the average between-group difference, but adjusted for covariates in the model

### Routine service level data

None available.

### Qualitative data

To be reported in a separate process evaluation.

### Economic analysis

#### Intervention delivery and costs

The resources needed to deliver IGR3 over the 9-month observation period included training Care Call staff and monthly IT costs (Table 3). Pedometers cost £2.20 per participant in IGR3. All participants received the Leicester Diabetes booklet at a cost of £1 per participant. Supervision of health coaching staff at Care Call cost £200 per participant over 9-months based on salary rates for 240 h for an Agenda for Change Band 7 manager and 1283 h for an Agenda for Change Band 2 administrator. The average fixed costs per participant were £201 in IGR2 and £439 in IGR3.

**Table 3:** Costs of training and online dashboard to support delivery of IGR3

Hitachi training (×3 2-h sessions)	*N*	Cost
Trainers		
IT project manager	1	£300
IT technical manager	1	£225
Service designer	1	£375
Trainees		
Care call service manager	1	£312
Diabetes specialist nurse	6	£1512
Health advisor	5	£900
Programme administrator	1	£138
Total training cost		£3,762
Training cost per participant in IGR3		£36
Online dashboard costs (per month, including VAT)		
Set up and hosting services		£930
Server rental		£410
Third line support		£1020
Total costs over 9 month trial		£21,240
Dashboard cost per participant in IGR3		£200

The average call time (including administration) for action planning was slightly higher in IGR3 compared with IGR2. Among those who had at least one attempted follow-up call the average call time (including administration) was similar in both groups (Table 4). Combining the call time (including administration) with unit costs of staff delivering the interventions, the mean cost of calls per participant was £144 (SD £44) for IGR2 and £142 (SD £56) for IGR3, excluding the seven withdrawals before action planning call attempts.

**Table 4:** Mean time in minutes for delivery of action planning and follow-up calls over 9-months by allocation group

	IGR2 (*n* = 99)		IGR3 (*n* = 93)	
	Mean (SD)	Range	Mean (SD)	Range
Action planning call				
Pre-call admin	3.8 (2.8)	0–15	5.6 (3.9)	0–24
Call time	28.1 (8.1)	2–47	28.3 (11.0)	5–88
Post call admin	20.1 (7.4)	6–45	21.0 (11.5)	4–76
Total	52.5 (13.7)	13–96	54.9 (20.7)	14–136
Follow-up calls				
Pre-call admin	6.6 (3.8)	0.7–20.0	6.8 (4.1)	0.0–20.0
Call time	21.4 (5.4)	11.0–45.5	21.9 (6.5)	4.0–50.0
Post call admin	12.4 (3.8)	5.7–28.0	13.0 (5.2)	5.7–40.0
Total	40.3 (10.2)	25.0–78.0	41.8 (11.4)	20.0–89.0

#### Cost effectiveness analysis with imputation

Table 5 presents the adjusted estimates of the incremental costs associated with delivery of interventions from a CCG perspective. IGR3 is associated with a mean incremental cost of £236 (95% CI £223 to £250) and a mean ICER of £182 (95% CI £84 to £931) per point improvement on the CSQ-8. The complete cases analysis showed a similar incremental costs per participant with a smaller difference in CSQ-8, leading to a much higher ICER with wider confidence interval (supplementary material 1).

**Table 5:** Cost-effectiveness analysis from clinical commissioning group perspective

Costs/outcomes	IGR2 (*N* = 103)	IGR3 (*N* = 106)
Cost of training, managing and other materials (mean, SE)	£201 (£0)	£439 (£0)
Cost of calls made within 9 months period (mean, SE)	£144 (£4)	£141 (£6)
Total intervention costs (mean, SE)	£345 (£4)	£581 (£6)
Unadjusted difference in intervention costs (mean, 95% CI)	£236 (£222 - £250))	
Adjusted difference in intervention costs^a^ (mean, 95% CI)	£236 (£223 - £250)	
CSQ-8 score (mean, SE)	27.1 (0.5)	28.5 (0.5)
Unadjusted difference in CSQ-8 score (mean, (95% CI)	1.3 (0.1–2.5)	
Adjusted difference in CSQ-8 score^a^ (mean, 95% CI)	1.3 (0.1–2.5)	
ICER (mean, 95% CI)	£182 (£84 - £931) per point improvement on CSQ-8	

*CI* confidence interval, *ICER* incremental cost-effectiveness ratio, *SE* standard error

^a^adjusted for age and gender

## Discussion

There was no evidence that participants who used a digitally-enabled telephone coachin intervention that included an online dashboard plus telephone health coaching for preventing diabetes were more satisfied with their healthcare than participants who used a telephone only health coaching intervention. While there was an indication that participants who received the digital intervention were more activated, had improved health status and reduced depressive symptoms, the differences compared with participants in the telephone only group were not significant. At follow-up participants in both groups had lower HbA1c and BMI but the between group difference for these outcomes was not significant. There were no significant differences between the groups for other secondary outcomes. Attrition was slightly higher in IGR3 and a quarter of participants in this group switched to IGR2 and received the telephone only intervention. However the per protocol analysis returned similar results to the intention-to-treat analysis suggesting that participants were satisfied with the overall health coaching approach regardless of allocation. Contact time between health coaches and participants was broadly equivalent in both groups. Although both were used for less than the maximum of 180 h allotted for the IGR pathway, IGR3 was not found to improve staff efficiency as expected. From a provider perspective the additional cost of the digital intervention was £236 ($332; €270) per participant to deliver, which consisted mostly of the training and platform costs of the online dashboard.

Previously it has been shown that satisfaction with telephone only health coaching is associated with higher levels of activation and number of sessions completed among those enrolled in a behaviour change programme to prevent diabetes.^24^ While there was a signal that participants in IGR3 were more satisfied and more activated than those in IGR2, we cannot be certain this was associated with the availability of the digitally-enabled intervention. Indeed, because there was no difference in uptake and number of follow-up telephone coaching calls between the two groups, it is still possible that any difference in satisfaction and activation may be attributed to the quality of health coaching.

While this trial was not set-up as a superiority test for clinical outcomes, it is instructive to compare before and after results achieved in CATFISH with those reported in comparable studies that tested lifestyle interventions for preventing diabetes in routine practice. In updating the review by Dunkley et al, Public Health England reported a pooled reduction in HbA1c of 0.07% for 11 pragmatic lifestyle interventions at 12–18 months follow-up.^25^ A similar result was reported at 6-months for a community based diabetes prevention programme run along pragmatic lines in the NHS.^26^ In CATFISH the overall performance of the IGR Care Call intervention was associated with a mean reduction in HbA1c of 0.10% with no difference between trial groups. This is equivalent to results of more intensive national diabetes prevention programmes such as those run in the United States which showed a mean reduction in HbA1c of 0.10% at 12 months.^8^ Similarly, weight loss results for the IGR Care Call interventions were equivalent or outperformed results from comparable pragmatic interventions such as Let’s Prevent which reported a mean difference of 0.11 in BMI scores between the intervention group and standard care.^26^ Larger effects were reported by Block et al who tested the effects of a fully automated digital behaviour change intervention among people with prediabetes.^19^ In that trial participants who used the web-based intervention reported a mean reduction in HbA1c of 0.26% and a mean reduction of 1.05 in BMI scores at 6-months. While participants were not supported by face-to-face or telephone coaching they did have access to social support via online networks and engaged in team based competitions suggesting that peer support and gamification might be critical to the success of online interventions.

Attrition is typically higher in trials of web-based interventions compared with controls, with drop-outs reported to be 22.5%.^27^ In CATFISH drop out from the web plus telephone group was 24.5% when withdrawals and those who switched to the telephone only group are combined. It is clear that therapeutic contact on the telephone can improve usage and outcomes of web-based interventions.^16^ However, unlike other web delivered lifestyle interventions that used telephone support we did not see a difference between the two groups in uptake of the telephone element of health coaching and we do not know if telephone coaching improved usage of the digital content of the intervention.^28^

This was not a cost-effectiveness study but we did assess costs attributed to the set-up and delivery of the digital intervention. The higher cost for using IGR3 were mainly associated with the web elements. The expected effect on efficiency was not found in the trial, which could be a result of lack of integration between the telephone and digital components. If the digital platform only served as a notebook, the platform could potentially only add additional burden associated with the training without any intended benefit. With no efficiency savings gained, it would be for the provider to judge if the additional cost per point of improvement on the CSQ-8 is worth paying. A key advantage of digitally-enabled interventions is that they have potential to reach economies of scale, which might take place as the user base grows. Based on modelling used in the digital pilot of the National Diabetes Prevention Programme in the NHS,^29^ the digitally-enabled telehealth intervention designed by Hitachi is purported to achieve efficiencies of scaling over telephone only interventions at 3000 users per year with the use of cloud based infrastructure and improved management capacity. In that model the cost of the digitally-enabled intervention would be £383 per person; a fully optimised cost basis is achievable at 10000 users per year costing £309 per person. There is potential to reduce the cost of the digitally-enabled intervention further with the use of a dynamic care pathway whereby users are segmented into three categories of health coaching support. In this dynamic model the cost per person is proposed to be £326 with a user base of 3000 per year, and £292 per person with a user base of 10000.

The digital intervention was embedded within an existing service that was delivered as part of routine care for people with NDH and in this sense the CATFISH trial responds to the need to address the translational gap between evidence and practice in diabetes prevention.^30^ Additionally the CATFISH trial evaluated end-user satisfaction as a primary outcome and thereby underscores the importance of assessing patient experience of interventions.^31^ As with most trials there is the possibility that we only reached and included a self-selecting cohort of eligible patients and that this group were the most motivated to engage with the intervention. However, participants were blind to treatment allocation and were not aware which intervention was novel; researchers and analysts were also blind to treatment allocation. Finally, drop outs from IGR3, including those who switched to IGR2, was moderately high but we do not know the reasons for this, nor do we know why once engaged in health coaching participants completed their call schedules. These questions will partly be addressed in a separate qualitative process evaluation.

In the context of continued roll out of diabetes prevention programmes, such as that launched by NHS England, it is important to recognise that relatively low intensity interventions such as the ones deployed in CATFISH have an important role to play in translating evidence into practice.^32^ Encouraging patients to engage with digital health interventions is a challenge and their reach and scaling are likely to hinge on successful outreach and enrolment activities.^33^ Where digital interventions are preferred it is critical that inclusion is enhanced to contend with higher rates of digital exclusion among the most deprived and among those who lack the confidence and skills to use online approaches.^34^ As with other digital interventions there is also need to consider how to maximise adherence and optimise usage and the use of games and SMS alerts, as well as improving accessibility on mobile platforms are possible avenues for further evaluation. Critical too is the need for a better understanding of the optimal combinations of behaviour change techniques and mode of delivery for web-based interventions. A review of 52 online health interventions showed that only usability was associated with effectiveness, but there was little evidence that pointed to the most effective combinations of behaviour change techniques.^35^ Component network meta-analysis is a relatively new approach to meta-analysis that can unpick the treatment effects of different components includes in complex composite interventions.^36^ There is scope for further elaboration of the most effective combinations of components for web-based behaviour change interventions using this approach. Definitive testing of candidate interventions would further enhance the evidence base for web-based behaviour change and health promotion interventions.

We showed that user satisfaction and experience of a digital-enabled telephone coaching intervention that included an online dashboard and telephone health coaching was broadly equivalent to that of a routinely delivered telephone-only coaching intervention for people with NDH. On the basis of user satisfaction there is scope to consider this digitally-enabled telephone coaching intervention as an option for decision makers with responsibility for commissioning diabetes prevention services. However there is still uncertainty about cost-effectiveness of this digitally-enabled telephone coaching intervention and further definitive evaluation is warranted. Furthermore, future work is needed to assess whether economies of scale are achievable outside the context of trial based evaluations where user bases are likely to be much larger.

## Methods

### Study design and participants

CATFISH was an individually randomised controlled trial conducted at the Salford NDH Care Call (previously known as IGR Care Call) service provided by Salford Royal NHS Foundation Trust, Greater Manchester. The trial protocol has been previously published.^37^ Ethical approval was granted by NHS Research Ethics Committee for the East of England (Cambridgeshire and Hertfordshire) (reference no 15/EE/0117).

Participants for CATFISH were identified from referrals from primary care or community teams to NDH Care Call. Referral criteria to NDH Care Call were: moderate or high risk on the Leicester Diabetes Risk Score Assessment Tool^38^ and HbA1c = 42–47 mmol/mol (6.0–6.4%) or a previous diagnosis of IGR with 1x confirmatory blood test (HbA1c within the previous 6 months).

Additional eligibility criteria for inclusion in CATFISH were adults aged >18 years and access to a telephone and home internet. We excluded people with a diagnosis of type 2 diabetes (HbA1c of ≥ 48 mmol/mol [≥6.5%]); a diagnosis of gestational diabetes; did not read or speak English; and those incapable of participating as indicated by their GP because of dementia, learning difficulties, vision or motor skills limitations, serious and enduring mental health problems.

### Randomisation and masking

Contact details of eligible patients were forwarded to the Care Call programme administrator who asked whether patients were willing to discuss participation in the CATFISH trial. Patients who verbally consented to be contacted about CATFISH were then telephoned by the research team (AB and EB) who checked their eligibility for the trial and invited them to agree to a baseline assessment. Once signed consent forms had been received and the baseline assessment had been completed participants were randomised using a central randomisation service provided by the Manchester Academic Health Science Clinical Trials Unit at the Christie NHS Foundation Trust. Participants were allocated 1:1 by minimisation using BMI and HbA1c at baseline. This technique ensures that treatment groups are very closely similar for several variables, even in small samples.^39^ The principal investigator and NDH programme administrator were aware of allocations; research staff and analysts were blinded to allocation. Treatment allocation was concealed from participants at the baseline assessment appointment. It was not possible to blind the health professionals delivering the interventions.

### Interventions

The telephone only service was known as IGR2. The digitally-enabled telephone service was known as IGR3. The service specification was adapted from the Diabetes Care Call service and a previously piloted 6 month telephone delivered lifestyle and education programme for people with impaired glucose tolerance.^12,40,41^ The core content for both IGR2 and IGR3 includes education about reducing the risk of developing diabetes, goal setting and action planning, and coaching from health advisors to support patients to achieve their goals and to maintain and review their action plans. Health advisors are NHS Agenda for Change band 4 workers who received at least two months in-house induction training that included the X-pert 6-week diabetes patient education programme which includes education sessions about the prevention of diabetes, and education sessions for newly diagnosed type 2 diabetes patients and insulin starters.^42^ Additionally all advisors completed a 1-day training course in motivational interviewing provided by Advancing Quality Alliance and delivered by an independent training company.^43^ Advisors were also given educational materials, took part in telephone role-play calls, observed diabetes clinics, attended medication training with the Diabetes Team, and shadowed calls from trained advisors.

The telephone schedule was similar for IGR2 and IGR3 and comprised an introductory call from the programme administrator, followed by a 30–40 min action planning phone call from a diabetes specialist nurse or dietician [call 1]; 6 × 10–20 min phone calls over six months from the health advisor [calls 2–7]; and a final step-down 10–20 min call at nine months [call 8]. All calls were outbound and heath advisors maintained contact with the same patients throughout the care pathway.

Before the action planning call all participants received a copy of the Leicester diabetes booklet. Participants completed ‘My Plan’ which helped them to reflect on how they might make changes to their food choices and activity to manage their risk of diabetes. The action planning call was not scripted and focused on explaining the significance of blood tests results, understanding risk factors for diabetes, exploring participants’ readiness to change and encouraging them to identify SMART goals. Weight loss targets of 5 and 10% were communicated to the participants. The six follow-up calls were guided by scripts modelled on those used by Diabetes Care Call but with an emphasis on diet and exercise and tailoring to ensure that call content was patient-centred and flexible to support their individual goals. The step-down call at 9 months provided an opportunity to negotiate and agree onward action plans and discharge participants back to GP care.

The critical innovation in IGR3 was the addition of an online dashboard that was accessible by participants and health advisors. The dashboard was co-designed with patient groups and front-line NHS Health Advisors, based on both Hitachi’s experience of delivering diabetes prevention and well-being programmes in Japan and SRFT experience of developing and delivering a telehealth diabetes prevention programme known as Diabetes Care Call.^12^ The dashboard was designed for use on desktop computers only. The patient view included an interactive and dynamic log that enabled participants to track their progress against action plans and monitor their weight, physical activity, and blood glucose. Additionally the dashboard view included a space for participants to message health advisors and a progress tracker to identify completed and future call events (Supplementary 2).

Before the action planning call, the participants allocated to IGR3 were sent a pedometer to log their activity during the six months exposure to the intervention. Participants allocated to IGR3 also completed an online self-assessment before the action planning call. The IGR3 care pathway was supported by three embedded videos on the online dashboard: Introduction; Guide to Self-Assessment; Guide to the Dashboard (supplementary 3). Health advisors could access logged information to assess the progress of the participants and tailor the follow-up calls accordingly.

### Primary outcome

The primary outcome was the mean difference in total score of the Client Satisfaction Questionnaire (CSQ-8)^44^ at 9 months post-randomisation. This end point was chosen to coincide with the timing of the scheduled step-down call. The CSQ-8 is an 8-item generic survey instrument used in primary care clinical trials and has been widely used as a measure of user satisfaction of web-based interventions.^45^ It is scored using a four-point Likert with a range from 8 to 32; higher scores indicate higher satisfaction. The CSQ-8 was measured only at follow-up with computed scores used to calculate the mean difference between groups.

### Secondary outcomes

#### HbA1c concentration

Glycated haemoglobin (HbA1c) was measured with a blood test taken from participants after the step-down call at the Barnes Clinical Research Facility, SRFT. Sample type and volume used were fluoride oxalate (yellow), 1 mL; reference range and units were 3.0 to 6.0 mmol/L. Participants who declined a blood test at follow-up were asked to consent to sharing the results of routine blood tests taken at their general practice. HbA1c represents average blood glucose over a 8 to 12 week period.^46^ We used routine test results that were reported between 12 weeks before or 12 weeks after the end of follow-up.

#### BMI

BMI (kg/m^2^) was measured using height (m) taken at baseline and weight (kg) measured at follow-up using ISO 9001:2008 standard scales (Seca model 8751) that were calibrated using weights traceable to the National Physics Laboratory Standards of Mass.

#### Quality of life

The five-item EQ-5D-5L is a generic measure of health-related quality of life consisting of the EQ-5D descriptive system and EQ Visual Analogue Scale (EQ VAS).^47^ The first part consists of 5 domains: mobility, self-management, ability to do usual activities, pain, anxiety and depression, with 5 levels of severity for each domain (no, slight, moderate, severe, and extreme problems). The VAS records an individual’s perceived self-rated health, ranging from 0 to 100; higher values indicate better general health. The EQ-5D-5L was used at baseline and follow-up.

#### Mental health

The Mental Health Inventory (MHI-5) is a 5-item scale which measures general mental health, including depression, anxiety, behavioural-emotional control and general positive affect.^48^ We used the recommended score ≤ 60 (high scores on this scale represent greater well-being) to indicate the presence of ‘probable depression’;^49^ continuous scores were used in the analysis. The MHI-5 was collected at baseline and at follow-up.

#### Health experience and self-management

We evaluated self-management behaviours using the revised version of the Summary of Diabetes Self-Care Activities (SDSCA) scale.^50^ The SDSCA includes items that assess diabetes self-management across core domains related to: general diet, specific diet, exercise, blood-glucose testing, foot care, and smoking. We used items relevant to diabetes prevention and assessed the number of days per week participants engaged in healthy and unhealthy behaviours (i.e., eating ≥5 portions of fruit and vegetables; eating high fat food; participating in ≥30 min of physical activity; participating in specific a specific exercise session; drinking alcohol). We also included an item on number of cigarettes smoked per day. Health experience and self-management data were collected at baseline and at follow-up.

#### Patient activation

The Patient Activation Measure (PAM)−13 is a self-report measure of patient knowledge, skills and confidence in self-management for long-term conditions.^51^ We used the short 13 item version.^52^ The score (0–100) can be used to segment patient populations into four levels of activation but we used the continuous score in the analysis. The PAM-13 was collected at baseline and at follow-up.

### Statistical analysis

We powered the study to have 90% power (with a 2-sided alpha level of 5%) to detect a standardised effect size of 0.5 on the CSQ-8. Allowing for a 15% attrition rate, 200 participants were needed in total (100 per group). Basic summary statistics (mean, SD, minimum, maximum) were calculated, by trial group, for each of our primary and secondary outcome variables. For the CSQ-8, data were only available at follow-up, whereas for all other outcomes, data were available at both baseline and follow-up. In order to formally test for post-intervention differences between CSQ-8 scores in the two groups, we conducted linear regression analysis, controlling for participant age-group, gender and baseline measurements of BMI, HbA1c, Patient Activation, MHI-5 and self-rated general health status. The primary analysis was conducted following the intention-to-treat principle. The CSQ-8 was only available for 181 observations whereas between 179 and 181 observations were available for secondary outcomes. The missing data in the outcome and covariates were handled by multiple imputation using chained equations, with 20 replications. To ease interpretation and to allow comparison with published studies, we estimated a standard effect size (Cohen’s d) for the CSQ-8 as the difference in follow-up means divided by the pooled baseline standard deviation for all participants. Missing data for secondary outcomes were not imputed but presented in available cases (Table 2). A per-protocol approach that analysed participants based on final treatment destination was used as a sensitivity analysis, again using the same multiple imputation approach as in the primary analysis. A secondary, complete-case, analysis was also conducted; here, we derived percentile-based, bootstrapped standard errors using 10,000 replications. Analyses were conducted using Stata Statistical Software: Release 15.

### Health economic analysis

The health economic analysis aimed to identify and measure the cost and benefits of delivering IGR2 and IGR3. The primary analysis was conducted from a Clinical Commissioning Perspective (CCG) perspective using intervention costs and the CSQ-8 to derive an additional cost per additional point of the CSQ-8. CCGs are clinically-led statutory NHS bodies responsible for the planning and commissioning of health care services for their local area. All costs were presented in UK pounds Sterling (£) for the financial year 2015–16. Costs were inflated to 2015–16 price levels where necessary using the Hospital and Community Health Services pay and price inflation index.^53^ No discount rate was applied to either costs or effectiveness because the follow-up period was 9 months.

#### Intervention costs

A micro-costing exercise was conducted following the methods of technology appraisal recommended by NICE.^54^ Training to support delivery of IGR3 was provided by Hitachi. It consisted of three 2-h sessions conducted by the IT project manager, IT technical manager and service designer. The Care Call service manager, programme administrator, six diabetes specialist nurses/diabetic dieticians and five health advisors attended the training. The cost of development of IGR3 platform was considered a ‘sunk cost’ and therefore not included.^55^ The total costs of the training were evenly allocated to the IGR3 participants.

The online dashboard was costed for the 9 months intervention period using contract pricing provided by Hitachi. This included monthly costs for set up and hosting services, server rental and third line support, including VAT. These fixed costs were evenly allocated to each participant in IGR3.

Unit costs of staff delivering both IGR2 and IGR3 were based on estimates of costs of health and social care compiled by the Personal Social Services Research Unit (PSSRU)^53^ (supplementary material 4). These unit costs were then multiplied by the time spent recorded in the call log or timesheet during the trial period to estimate the costs of delivering the interventions.

#### Cost-effectiveness

Incremental cost-effectiveness analysis combined the intervention costs with the primary outcome to generate an incremental cost-effectiveness ratio (ICER), by dividing the mean difference in costs between the two trial groups by the mean difference in effect.^56^ From a CCG perspective the ICER was interpreted as the additional intervention cost per participant for one point improvement on CSQ-8.

Missing data were handled by multiple imputation following Rubin’s rules,^57^ assuming that any missing data were missing at random. A chained equation model was developed and predictive mean matching by intervention groups was used as the imputation method, using the ten nearest neighbours to the prediction as a set to draw from. The number of imputations was set to approximately the highest percentage of missing data in all variables included in the imputation model.^58^ Due to the non-normal distribution of both cost and outcome data, 5000 replacement samplings were generated using bootstrap technique to derive 95% confidence intervals. A sensitivity analysis was undertaken to repeat the CEA using complete cases.

## Supplementary information

Supplementary material tables combined

Supplementary material 2

Supplementary material 3

## Data Availability

The data that support the findings of this study are available from the corresponding author upon reasonable request.

## **Supplementary information**

**Supplementary information** accompanies the paper on the *npj Digital Medicine* website (10.1038/s41746-019-0080-6).
