# Supplementary material for: Satisfaction with a digitally-enabled telephone health coaching intervention for people with non-diabetic hyperglycaemia
Source: NPJ Digit Med. 2019 Feb 4;2:5. doi: 10.1038/s41746-019-0080-6 (PMC6550206; doi:10.1038/s41746-019-0080-6)
Supplement: Supplementary file 1 — Supplementary material tables combined [file 41746_2019_80_MOESM1_ESM.docx]

Supplementary material 1: Cost-effectiveness (complete cases) analysis from a clinical commissioning group perspective

| Costs/Outcomes | IGR2 (N=50) | IGR3 (N=55) |
| --- | --- | --- |
| Cost of training, managing and other materials (mean, SD) | £201 (£0) | £439 (£0) |
| Cost of calls made within 9 months period (mean, SD) | £147 (£41) | £140 (£58) |
| Total intervention costs (mean, SD) | £348 (£41) | £580 (£58) |
| Unadjusted difference in intervention costs (mean, 95% CI) | £231 (£212 - £251) | |
| Adjusted difference in intervention costs^a^ (mean, 95% CI) | £230 (£212 - £249) | |
| CSQ-8 score (mean, SD) | 27.0 (5.7) | 27.7 (5.0) |
| Unadjusted difference in CSQ-8 score (mean, 95% CI) | 0.6 (-1.4 – 2.7) | |
| Adjusted difference in CSQ-8 score^a^ (mean, 95% CI) | 0.2 (-1.8 – 2.5) | |
| ICER (mean, 95% CI) | £1,150 (-£3,618 − £3,203) per point improvement on CSQ-8 | |

^a^ adjusted for age and gender

CI: confidence interval; ICER: incremental cost-effectiveness ratio; SD: standard deviation

Supplementary material 4: Unit cost of staff delivering and managing the intervention

| Item | Unit cost | Source | Details |
| --- | --- | --- | --- |
| Health coach | £30 per hour | Unit costs of health and social care (PSSRU) | Scientific and professional staff (band 4) |
| Diabetes specialist nurse/Diabetic dietician | £42 per hour | Unit costs of health and social care (PSSRU) | Scientific and professional staff (band 6)/ nurse band 5/6 |
| Service manager | £52 per hour | Unit costs of health and social care (PSSRU) | Scientific and professional staff (band 7) |
| Programme administrator | £23 per hour | Unit costs of health and social care (PSSRU) | Scientific and professional staff (band2) |

PSSRU: Personal Social Services Research Unit
