# Supplementary figures and images for: Satisfaction with a digitally-enabled telephone health coaching intervention for people with non-diabetic hyperglycaemia
Source: NPJ Digit Med. 2019 Feb 4;2:5. doi: 10.1038/s41746-019-0080-6 (PMC6550206; doi:10.1038/s41746-019-0080-6)

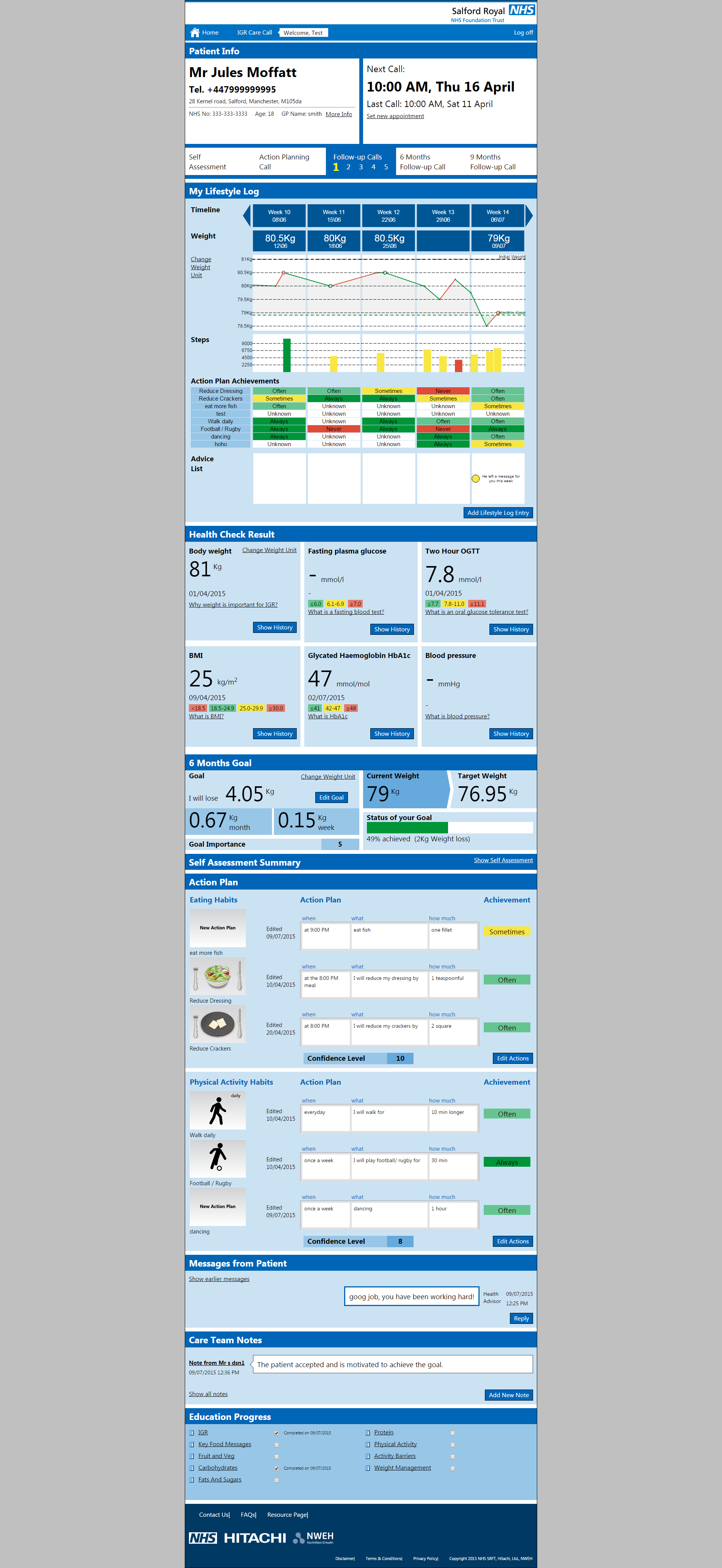

Supplement: Supplementary file 2 — Supplementary material 2 [file 41746_2019_80_MOESM2_ESM.png]

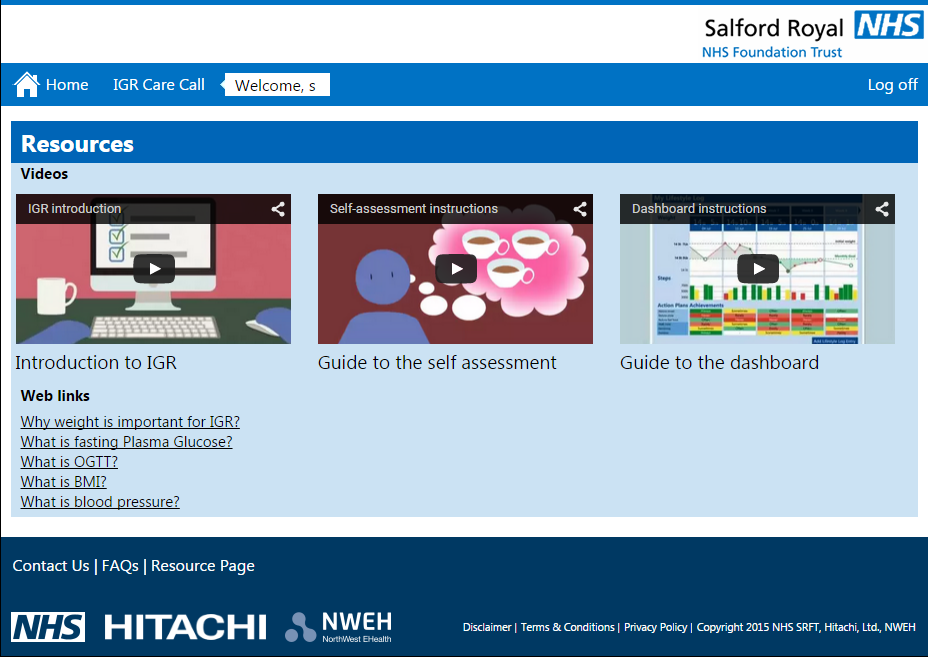

Supplement: Supplementary file 3 — Supplementary material 3 [file 41746_2019_80_MOESM3_ESM.png]
